# The impact of heavy metal concentrations on aquatic insect populations in the Asan Wetland of Dehradun, Uttarakhand

**DOI:** 10.1038/s41598-024-52522-5

**Published:** 2024-02-27

**Authors:** Sazia Tabassum, C. B. Kotnala, Mohammed Salman, Mohd Tariq, Afzal Husain Khan, Nadeem A. Khan

**Affiliations:** 1 Ecology Lab, Department of Zoology, HNB Garhwal University (A Central University), BGR Campus Pauri Garhwal, Pauri, Uttarakhand 246001 India; 2 Department of Civil Engineering, Indian Institute of Technology, Roorkee, Haridwar, 247667 India; 3 Department of Civil Engineering, College of Engineering, Jazan University, Jazan, Saudi Arabia; 4 Department of Civil Engineering, Mewat Engineering College, Nuh, Haryana, 122017 India

**Keywords:** Ecology, Energy science and technology

## Abstract

This study, centered on the Asan Wetland in Uttarakhand, examines the ecological impact of heavy metals on aquatic insects biodiversity. It highlights the detrimental effects of metals like chromium, mercury, and lead, stemming from natural and anthropogenic sources, on aquatic insects diversity. Aquatic insects, particularly sensitive to water quality, are emphasized as key indicators of environmental health, illustrating the importance of understanding and managing the influences on wetland ecosystems. Wetland ecosystems are vulnerable to various environmental stressors, including pollution from heavy metals. These toxic substances can alter water quality parameters, disrupt nutrient cycling, and negatively impact the biodiversity and ecological balance of the system. This study aimed to evaluate the impact of several heavy metals (namely Cd, As, Cu, Fe, Pb, Ni, Zn, Al, Cr) on the distribution and biodiversity of various aquatic insect species, including Coeleoptera, Diptera, Ephemeroptera, Odonata, Plecoptera, and Trichoptera. The research utilized data collected between November 2021 and October 2022 from specifically chosen sites (S1, S2, S3) within the Asan Wetland in Dehradun, Uttarakhand. After collecting and identifying samples, various statistical (Sorenson, Shannon-Weiner diversity index, Margelef index) and multivariate tests (CCA, PCA, One-way Anova), have been applied to show the effects of these parameters. This study offers significant findings regarding the distribution patterns of heavy metals, the abundance of aquatic insects, and their interconnectedness within the ecosystem of the Asan Wetland. The abundance of aquatic insects, represented by 13 genera belonging to 6 orders, was assessed at three different sites (S1, S2, and S3) within the wetland. It was concluded that the heavy metals concentration and aquatic insects’ density increases and decreases vice-versa in monsoon and winter seasons might be due to unfavourable factors. These findings contribute to the understanding of ecological dynamics and potential impacts of heavy metals on aquatic biota in wetland environments.

## Introduction

Heavy metals are naturally occurring elements, but their presence in aquatic ecosystems is often amplified by anthropogenic activities such as industrial processes, mining, and agriculture. These metals are introduced into the environment through a variety of sources, including geological breakdown of rocks and minerals, industrial extraction and refining of ores, and metal utilization in various applications. The seepage of metals from waste disposal sites and the use of metal-based compounds in agriculture contributes significantly to this pollution^1,2^. Once introduced into aquatic environments, heavy metals like chromium, mercury, lead, cadmium, and arsenic exhibit high toxicity. They lead to adverse consequences for aquatic life, disrupting microbial ecosystems and causing physiological stress in plants. This includes chlorosis, impeded growth, reduced biomass production, and the generation of free radicals that damage plant structures^1,3^. These impacts highlight the need for a deeper understanding of heavy metal pollution in aquatic systems.

The toxicity of heavy metals, even at low concentrations, poses significant risks to living organisms. Metals like chromium, mercury, lead, cadmium, and arsenic are particularly harmful, impacting the distribution and population of macroinvertebrates, including aquatic insects^4^. These insects, as integral components of freshwater ecosystems, play critical roles in nutrient cycling, energy transfer, and maintaining food webs. They are highly sensitive to changes in water quality and are often utilized as bioindicators of environmental stress. Globally, aquatic insects are diverse, with approximately 45,000 species identified, and in India, around 5,000 species are found within inland wetlands^5,6^.

Studies across various continents have demonstrated how substrate types significantly influence the composition of aquatic fauna. Erosional habitats, shaped by water currents, generally harbor a richer diversity of species compared to sandy or silty environments, which typically support less diverse communities^7–9^. This variation in fauna composition is an important indicator of ecosystem health. For instance, the presence of stoneflies in a stream indicates good water quality, whereas dominance of worm-like organisms points to potential pollution issues^10,11^.

Human activities, notably industrial effluents, agriculture, and urbanization, have considerably impacted freshwater ecosystems^12,13^. Research indicates a notable correlation between the presence of specific aquatic insects and levels of heavy metal contamination^14^. In regions like Uttarakhand, with its rich biodiversity and wetland ecosystems, understanding these impacts is critical for the conservation and management of freshwater environments^15,16^. Uttarakhand, nestled in the Himalayas, is abundant in natural resources and biodiversity, with numerous rivers, glaciers, and wetlands. This region has been the focus of various studies exploring the relationship between physicochemical parameters and aquatic life, particularly benthic organisms. Researchers have contributed significantly to our understanding of water ecology in these habitats, shedding light on the population structures and diversity of aquatic insects like Ephemeropterans^17,18^. Recent studies in the Doon Valley, encompassing aquatic, avian ecology, and plant diversity, particularly in the Asan wetland, have expanded this knowledge base^19–23^. Only a limited number of studies have been conducted to investigate the impact of heavy metals on aquatic insects. As^24^ states, heavy metal pollution can lead to various effects on these organisms. These studies underline the importance of examining the impact of environmental stressors, such as heavy metal pollution, on aquatic ecosystems. Our study aims to explore the impact of heavy metal (namely Cd, Pb, Cu, Zn, Ni, Fe, As, Al)pollution on aquatic insects, specifically the Ephemeroptera, Plecoptera, Trichoptera, and Odonata groups, in the Asan Wetland of Dehradun, Uttarakhand. Utilizing multivariate analysis, it seeks to understand the complex relationships between heavy metal concentrations and the population dynamics of these vital insect species.

## Study area

**Figure 1:**
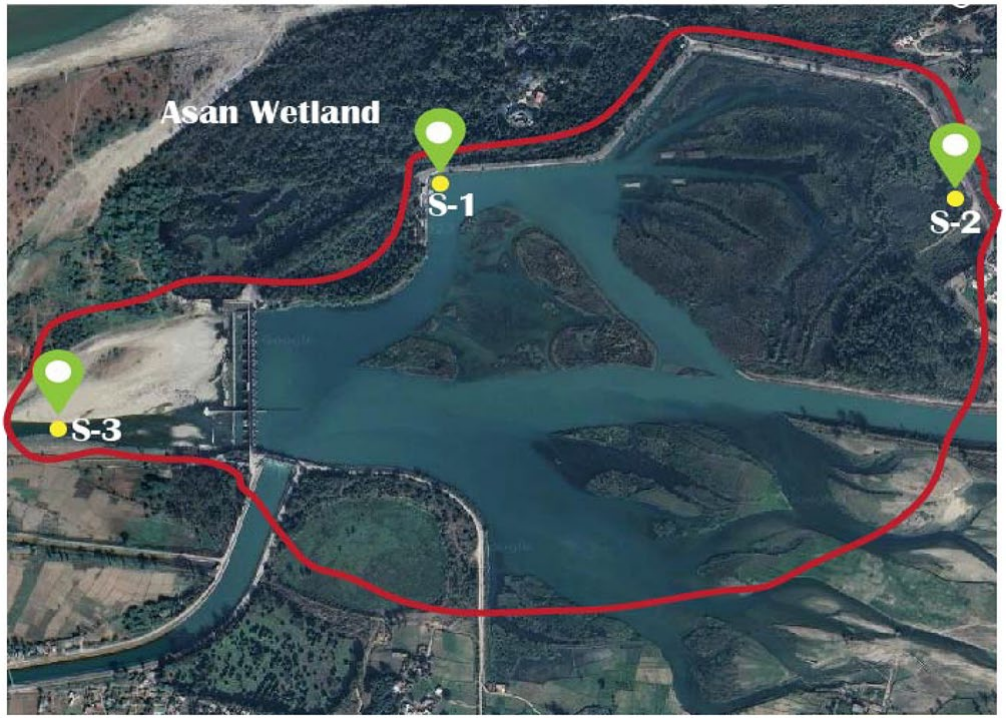
Map showing study sites (S1, S2, S3) in the Asan wetland. The yellow dot symbol indicates sampling stations. (Satellite imagery source: Google Earth Map; https://earth.google.com/).

Following the construction of the barrage in 1967 at the confluence of the Asan River and the outlet channel of the Dhalipur power station, the Asan wetland has been officially designated as the Asan Conservation Reserve. Located in the central Himalayas Doon valley, the barrage spans a length of 287.5 m and occupies an area of 3.2 square kilometers. The wetland itself is encompassed by dense Sal Forest, cultivated and pastured lands, the Rampur Forest Block, mixed forests, and land owned by the irrigation department. It comprises both shallow and deep-water regions. Asan River, a significant tributary of the Yamuna River, flows through the wetland, and the western side of the wetland features the aforementioned 287.5-m-long barrage, with a water level maintained at 403.3 m above sea level. The area experiences a rainy season that receives an average rainfall ranging from 250 to 275 cm. The Asan wetland serves as a natural habitat for migratory as well as local aquatic bird species, including waterfowl, waders, and divers. Due to its rich biodiversity and ecological sensitivity, the Asan wetland is recognized as one of the biodiversity hotspots and environmentally significant habitats in the Doon valley. Therefore, the Uttarakhand government (India) has declared it as a conservation reserve. The wetland has a total catchment area of 1600 square kilometers, with contributions from both the Asan and Yamuna rivers. In the case of the Asan Wetland in Dehradun, Uttarakhand, the increase in heavy metal concentration, particularly, can be attributed to several factors. These include agricultural runoff, which often contains pesticides that are a primary source of heavy metals^25^. Other contributing factors are soil erosion, industrial discharges, sediment transport, and poor waste management practices, all of which contribute to river water pollution and subsequently affect the wetland^25,26^. Figure 1 and Table 1 shows selected sampling sites and their description, respectively in the Asan wetland of the Doon valley. The decision to limit the number of sampling sites to three have been influenced by logistical, financial, or geographical considerations, ensuring that the study was manageable and that the sites selected provided a comprehensive overview of the wetland's condition. This targeted approach is a common practice in environmental studies, where researchers often select a limited number of representative sites to obtain a balanced view of the ecosystem being studied^27,28^.

**Table 1:** Showing description of sampling sites (S1, S2, S3).

S. No	Sampling sites	ID No	Description
1	Site-1	S-1	GMVN Asan conservation resort
2	Site-2	S-2	Confluence of reservoir and Yamuna canal
3	Site-3	S-3	Asan Barrage bird sanctuary

### Health risks associated with bioaccumulation of heavy metals

Regarding studies on heavy metal contamination in the Asan Wetland of Dehradun, Uttarakhand, and its potential health risks, there appears to be limited direct research specific to this area. However, general research on heavy metal contamination in wetlands and its effects can provide insights.

For instance, a study on heavy metal contamination in wetland sediments highlights the potential carcinogenic health hazards from such contamination. This research shows that heavy metals like Pb and Cr in sediments can pose significant health risks, including cancer, especially when these metals enter the food chain through the consumption of fish, rice, or other foods grown in or near these wetlands^29^.

Health Risks from Consuming Contaminated Fish: Another study conducted in a hazardous-waste contaminated wetland in Louisiana, USA, assessed the potential health risks associated with ingesting heavy metals in fish. This research, along with similar studies in Colombia, Mississippi River, and Bangladesh, collectively point to the significant health implications of consuming fish contaminated with heavy metals. These studies cover a range of heavy metals, including cadmium and lead, and their accumulation in fish tissues, indicating the pervasive nature of this issue across different ecosystems^30^.

While this study is not specific to the Asan Wetland, it underscores the importance of assessing heavy metal levels in wetlands and the potential health risks associated with consuming aquatic life from these areas. The findings from such studies are crucial for understanding the risks in any wetland area, including the Asan Wetland, where heavy metal contamination might be a concern. Monitoring and management of these environments are essential to mitigate the hazards to both the ecosystem and human health.

## Material and methods

### Sampling procedure

- **Sample Collection of Water**: The study in the Asan wetland involved meticulous water sampling at three designated sites (detailed in Table 1), conducted monthly from November 2021 to October 2022. For each sampling, one liter of water was collected and subsequently refrigerated for thorough analysis. The analytical focus was on detecting concentrations of various heavy metals, including Cadmium, Copper, Arsenic, Iron, Lead, Chromium, Zinc, Nickel, and Aluminium. These determinations were carried out using Inductively Coupled Plasma-Mass Spectroscopy (ICP-MS), following the precise methodologies outlined in the APHA 2012 guidelines. This technique ensures accurate quantification of each metal, crucial for assessing their impact on the wetland's aquatic life and ecosystem.
- **Collection and Preservation of Macroinvertebrates**: Aquatic insects from the benthic region were collected using a dip net from various sampling sites, including aquatic vegetation, debris, and submerged objects. Macroinvertebrates were also handpicked from macrophytes in the sampling area. The collected samples were examined, sorted with fine forceps, and preserved in 4% formalin. The collection methodology involved taking samples from a 1 ft. square area and then converting the values to 1 m square by multiplying with 10.764^9,23^.
- **Identification of Samples**: Upon collection, the macroinvertebrate samples underwent detailed analysis under microscopes (either dissected or stereozoom) with a minimum magnification of 10X. The identification process was comprehensive, considering various anatomical features such as gills, body segments, head shape, and appendages. This analysis utilized taxonomic keys developed by field experts, ensuring precise identification up to the genera level^9,32^. This detailed scrutiny is crucial for understanding the diversity and ecological roles of these macroinvertebrates in the wetland ecosystem.

## Statistical analysis

The data obtained from the study on heavy metals and aquatic insects was analyzed separately for each month using statistical techniques. Descriptive statistical analysis was performed to summarize the results, including measures such as average, minimum, maximum, and standard deviation. To examine the relationship between the biotic (aquatic insects) and abiotic (heavy metals) variables, the similarity coefficient (Sorensen index) was calculated using S = 2C/A + B (where A represents the taxa in sample A, B is the taxa in sample B, and C is the taxa in both samples) to assess the similarity of taxa across different months. The species diversity was estimated using the Shannon–Wiener diversity index (H′ = − Σpi.lnpi), which takes into account both the number of species present and the evenness of their distribution. The Margelef index was also computed to measure the evenness and show how close the number of individuals of a taxon during different months.

## Multivariate statistical analysis

The multivariate analysis conducted in this study was performed using the PAST software. To examine the hierarchical grouping among various orders of aquatic insects, multivariate cluster analysis (CA) was employed. This method entails grouping taxa into clusters based on their similarity within a particular category and their dissimilarity across different categories by applying CA, similar sites of aquatic insect taxa were identified and categorized, enabling a comprehensive understanding of their taxonomic relationships and patterns of similarity within and between different insect order^33^.

The canonical correspondence analysis (CCA) was employed to determine the adverse effect of heavy metals on the benthic population structure. CCA is a statistical technique used to explore the relationship between environmental variables and species composition. By identifying the main ecological factors that influence benthic populations, this analysis can provide valuable information for the management and conservation of aquatic ecosystems^21,22^.

Moreover, the study incorporated Principal Component Analysis (PCA) as a method to extract crucial parameters while minimizing information loss. The PCA was employed specifically to assess the variations observed in aquatic insects across diverse sites, facilitating the categorization and identification of potential sources related to trace metals. By utilizing PCA, the analysis aimed to gain insights into the relationships between trace metals and the distribution patterns of aquatic insects, thus facilitating the identification and characterization of potential contributing factors. The PCA served as a valuable tool in evaluating the variations among aquatic insect populations and elucidating the potential sources of trace metal contamination.

## Results

Heavy metal contamination was detected across all sites within the Asan Wetland. To provide a comprehensive overview of the variations in heavy metal parameters, the average monthly values and standard deviations for the year 2020–2021 at three specific sites (S1, S2, S3) are presented in Table 2. This data provides valuable insights into the temporal fluctuations and dispersion of heavy metal concentrations within the Asan Wetland, allowing for a better understanding of the overall contamination levels and potential ecological implications. The recorded parameters consistently showed higher average values during the monsoon season, followed by summer, and lower values during winter. During this period, it was found that the maximum values of cadmium (0.0853 ± 0.0457), copper (0.192 ± 0.168), arsenic (1.03 ± 0.0783), and Lead (0.576 ± 0.173) in the month of August and minimum values Cd (0.0023 ± 0.00057), Cu (0.0047 ± 0.00723), As (0.0053 ± 0.0058), Pb (0.006 ± 0.004) in January. Iron was maximum (5.74 ± 1.55) in the month of September and minimum (1.41 ± 1.98) in the month of January. Chromium was maximum (0.686 ± 0.018) in the month of May and minimum (0.01. ± 0.0012) in the month of January. Zinc was maximum (0.259) in the month of October and minimum (0.1 ± 0.080) in the month of April. Nickel and Aluminium was maximum (0.312 ± 0.232), (2.44 ± 0.459) in the month of September and minimum (0.007 ± 0.003), (0.044 ± 0.046) in the month of January, respectively like Iron. Table 3 in the study presents the average monthly variations of aquatic insects across different genera at three sampling sites (S1, S2, S3) in the Asan wetland, spanning from November 2021 to October 2022. The data is organized in a tabular format, listing various orders and genera of insects, such as Coleoptera (Cybister spp.), Diptera (Dineutes spp., Chironomous spp., Culex spp.), Ephemeroptera (Tabanus, Baetis spp.), and others. Each genus is associated with monthly counts, presented as mean values with standard deviations (e.g., 14.4 ± 16.4). The table reveals fluctuating population densities throughout the year, highlighting seasonal patterns in the presence and abundance of different insect groups in the wetland ecosystem.

**Table 2:** Average Monthly variations in the heavy metal parameters at S1, S2, S3 of the Asan wetland during the study from November 2021 to October 2022.

Heavy metals (ppm)	Cadmium	Copper	Arsenic	Iron	Lead	Chromium	Zinc	Nickel	Aluminum
NOV	0.0020 ± 0.0026	0.0067 ± 0.0072	0.0183 ± 0.0213	3.0700 ± 2.3300	0.0450 ± 0.0660	0.0650 ± 0.0470	0.2710 ± 0.0820	0.0970 ± 0.0825	0.8220 ± 1.0000
DEC	0.0020 ± 0.0014	0.0127 ± 0.0126	0.0126 ± 0.0132	1.9900 ± 2.7200	0.0570 ± 0.0517	0.0350 ± 0.0210	0.2060 ± 0.0620	0.0090 ± 0.0037	0.1600 ± 0.0540
JAN	0.0023 ± 0.0005	0.0047 ± 0.0072	0.0053 ± 0.0058	1.4100 ± 1.9800	0.0060 ± 0.0040	0.0100 ± 0.0012	0.1680 ± 0.0950	0.0070 ± 0.0034	0.0440 ± 0.0460
FEB	0.0033 ± 0.0011	0.0097 ± 0.0072	0.0066 ± 0.0021	1.9100 ± 1.7900	0.0230 ± 0.0151	0.0360 ± 0.0080	0.1140 ± 0.0700	0.0176 ± 0.0142	0.1820 ± 0.1600
MARCH	0.0036 ± 0.0023	0.0113 ± 0.0068	0.0063 ± 0.0016	2.0800 ± 1.9700	0.0420 ± 0.0192	0.0490 ± 0.0190	0.1120 ± 0.0250	0.0156 ± 0.0090	0.3620 ± 0.2710
APRIL	0.0390 ± 0.0494	0.0180 ± 0.0052	0.0066 ± 0.0006	2.0300 ± 1.3500	0.0660 ± 0.0550	0.3110 ± 0.4490	0.1000 ± 0.0800	0.0116 ± 0.0028	0.8170 ± 0.5030
MAY	0.0403 ± 0.0196	0.0480 ± 0.0269	0.0070 ± 0.0017	2.1400 ± 1.3900	0.0126 ± 0.1840	0.6860 ± 0.0180	0.1030 ± 0.0790	0.0136 ± 0.0025	1.1130 ± 0.7330
JUNE	0.0583 ± 0.0342	0.1210 ± 0.1004	0.2460 ± 0.2776	2.7100 ± 0.8600	0.2120 ± 0.2160	0.4280 ± 0.4880	0.1403 ± 0.0490	0.0153 ± 0.0020	1.4350 ± 0.7300
JULY	0.0136 ± 0.0049	0.0570 ± 0.0226	0.9180 ± 0.0104	2.6800 ± 0.6500	0.2370 ± 0.0830	0.1540 ± 0.0440	0.1843 ± 0.0570	0.0173 ± 0.0020	1.6010 ± 0.7110
AUG	0.0853 ± 0.0457	0.1920 ± 0.1680	1.0300 ± 0.0783	4.3200 ± 0.8500	0.5760 ± 0.1730	0.3500 ± 0.2780	0.2143 ± 0.0180	0.1490 ± 0.0771	2.1500 ± 0.8030
SEP	0.0536 ± 0.0275	0.0840 ± 0.0263	0.7420 ± 0.5590	5.7400 ± 1.5500	0.5260 ± 0.7260	0.4750 ± 0.2750	0.1720 ± 0.0620	0.3120 ± 0.2320	2.4400 ± 0.4590
OCT	0.0056 ± 0.0072	0.0090 ± 0.0068	0.0330 ± 0.0390	5.3000 ± 1.2100	0.0110 ± 0.0050	0.1610 ± 0.1140	0.2590 ± 0.0980	0.0170 ± 0.0040	2.2850 ± 1.6610

**Table 3:** Average Monthly variations of the aquatic insects at S1, S2, S3 of the Asan wetland during the study from November 2021 to October 2022.

ORDER/GENERA												
COELEOPTERA	N	D	J	F	M	A	M	J	JU	A	S	O
Cybister spp	14.4 ± 16.4	14.4 ± 12.4	25.1 ± 16.4	0.0 ± 0.0	10.8 ± 0.0	3.6 ± 6.2	0.0 ± 0.0	0.0 ± 0.0	3.6 ± 6.2	14.4 ± 6.2	14.4 ± 6.2	14.4 ± 6.2
DIPTERA												
Dineutes spp	35.9 ± 6.2	50.2 ± 6.2	57.4 ± 6.2	35.9 ± 6.2	21.5 ± 0.0	14.4 ± 12.4	10.8 ± 0.0	3.6 ± 6.2	0.0 ± 0.0	3.6 ± 6.2	14.4 ± 6.2	21.5 ± 4.35
Chironomous spp	7.2 ± 12.4	10.8 ± 18.6	28.7 ± 12.4	21.5 ± 10.8	14.4 ± 16.4	7.2 ± 6.2	0.0 ± 0.0	3.6 ± 6.2	0.0 ± 0.0	0.0 ± 0.0	7.2 ± 6.2	3.6 ± 6.2
Culex spp	35.9 ± 6.2	39.5 ± 12.4	43.1 ± 6.2	28.7 ± 16.4	25.1 ± 16.4	7.2 ± 12.4	7.2 ± 12.4	25.1 ± 6.2	28.7 ± 6.2	17.9 ± 6.2	14.4 ± 6.2	28.7 ± 16.4
EPHEMEROPTERA												
Tabanus	28.7 ± 12.4	39.5 ± 16.4	35.9 ± 12.4	21.5 ± 10.8	14.4 ± 12.4	14.4 ± 6.2	10.8 ± 0.0	3.6 ± 6.2	7.2 ± 6.2	3.6 ± 6.2	0.0 ± 0.0	10.8 ± 10.7
Baetis spp	57.4 ± 12.4	68.2 ± 22.4	82.5 ± 12.4	50.2 ± 16.4	32.3 ± 10.8	28.7 ± 6.2	17.9 ± 6.2	7.2 ± 6.2	0.0 ± 0.0	0.0 ± 0.0	10.8 ± 10.8	39.5 ± 16.4
ODONATA												
Heptogenia	64.6 ± 28.5	82.5 ± 34.6	104.1 ± 28.5	68.2 ± 32.9	53.8 ± 32.3	46.6 ± 22.4	28.7 ± 6.2	25.1 ± 27.1	32.3 ± 28.5	7.2 ± 12.4	21.5 ± 28.5	43.1 ± 28.4
TRICHOPTERA												
Crocothermis spp	39.5 ± 12.4	46.6 ± 16.4	25.1 ± 12.4	28.7 ± 27.1	35.9 ± 22.4	35.9 ± 22.4	35.9 ± 12.4	32.3 ± 10.8	21.5 ± 10.8	21.5 ± 18.6	21.5 ± 18.6	25.1 ± 22.4
Chimmarra	21.5 ± 21.5	39.5 ± 6.2	53.8 ± 21.5	25.1 ± 27.1	28.7 ± 16.4	14.4 ± 16.4	21.5 ± 18.6	10.8 ± 10.8	3.6 ± 6.2	3.6 ± 6.2	10.8 ± 10.8	21.5 ± 10.7
Molanna	17.9 ± 16.4	14.4 ± 16.4	21.5 ± 16.4	7.2 ± 12.4	14.4 ± 6.2	3.6 ± 6.2	3.6 ± 6.2	7.2 ± 6.2	3.6 ± 6.2	0.0 ± 0.0	10.8 ± 10.8	17.9 ± 16.4
Hydropsyche	35.9 ± 12.4	46.6 ± 12.4	61.0 ± 12.4	43.1 ± 10.8	17.9 ± 16.4	21.5 ± 10.8	17.9 ± 6.2	10.8 ± 0.0	3.6 ± 6.2	0.0 ± 0.0	14.4 ± 12.4	21.5 ± 10.7
PLECOPTERA												
Hydroptilla	25.1 ± 6.2	39.5 ± 6.2	39.5 ± 6.2	25.1 ± 6.2	14.4 ± 12.4	14.4 ± 6.2	10.8 ± 0.0	0.0 ± 0.0	0.0 ± 0.0	0.0 ± 0.0	7.2 ± 6.2	10.8 ± 2.17
Perla	14.4 ± 6.2	25.1 ± 6.2	35.9 ± 6.2	17.9 ± 6.2	14.4 ± 6.2	3.6 ± 6.2	10.8 ± 0.0	7.2 ± 6.2	0.0 ± 0.0	0.0 ± 0.0	7.2 ± 6.2	7.2 ± 6.2
Total	398.3 ± 169.9	516.7 ± 187.1	613.5 ± 270.7	373.2 ± 183.3	297.8 ± 168.5	215.3 ± 140.4	193.75 ± 175.8	136.3 ± 92.1	104.1 ± 76.5	71.8 ± 62.1	154.3 ± 129.1	265.5 ± 151.1

The overall average dissimilarity of aquatic insects of the Asan wetland at S1, S2, S3 during the study period (November 2021 to October 2022) is represented in Table 4. The mean of average dissimilarity of aquatic insects at all selected sites of Asan wetland was 23.3%, whereas the average dissimilarity in abundance was 22.17% as indicated by the multivariate Simpler test. This table also shows the mean and abundance of aquatic insects at three different sites.

**Table 4:** Average dissimilarity of aquatic insects of the Asan wetland at S1, S2, S3 during study from November 2021 to October 2022.

Taxon	Av. Dissim Mean	Av. Dissim abundance	Contrib. %	Cumulative %	Mean S1	Abundance S1	Mean S2	Abundance S2	Mean S3	Abundance S3
Heptogenia spp	7.259	6.858	30.94	30.94	257	257	641	641	883	883
Chimmarra spp	2.315	2.296	10.36	41.3	163	163	408	408	205	205
Baetis spp	2.098	2.016	9.094	50.39	315	315	501	501	420	420
Hydroptilla spp	2.001	1.942	8.761	59.15	210	210	385	385	323	323
Chironomous spp	1.858	1.636	7.383	66.54	35	35	105	105	194	194
Crocothermis spp	1.712	1.587	7.16	73.7	315	315	396	396	463	463
Culex spp	1.489	1.258	5.676	79.37	268	268	303	303	398	398
Tabanus spp	1.241	1.106	4.989	84.36	152	152	175	175	269	269
Molanna	1.132	1.052	4.748	89.11	70	70	140	140	161	161
Hydropsyche	0.943	0.9362	4.224	93.33	163	163	268	268	161	161
Perla spp	0.593	0.5849	2.639	95.97	163	163	175	175	108	108
Cybister spp	0.4869	0.5015	2.262	98.23	128	128	93.3	93.3	151	151
Dineutes spp	0.2066	0.3914	1.766	100	280	280	303	303	258	258
Overall average dissimilarity	23.3%	22.17%								

Sorensen similarity index of Aquatic insects between different months in the Asan wetland at S1, S2 and S3, during 2021–2022 is shown in Table 5. Maximum similarity was found between the winter months at all sites of the study. One way ANOVA of Aquatic Insects at S1, S2, S3 in the Asan wetland during the study period (November 2021–October 2022) is presented in Table 6. The *p*-value thus obtained (*p* = 0.155) represented that there is no significant difference between the three sites in the density of aquatic insects.

**Table 5:** Sorensen similarity index of Aquatic insects between different months in the Asan wetland at S1, during 2021–2022.

	N	D	J	F	M	A	M	J	JU	A	S	O
N	1.000	0.957	0.818	0.909	0.800	0.842	0.909	0.667	0.429	0.429	0.800	1.000
D		1.000	0.870	0.870	0.857	0.800	0.957	0.737	0.400	0.400	0.762	0.957
J			1.000	0.909	0.700	0.737	0.818	0.556	0.143	0.143	0.700	0.818
F				1.000	0.700	0.737	0.818	0.556	0.286	0.286	0.800	0.909
M					1.000	0.588	0.800	0.875	0.333	0.333	0.667	0.800
A						1.000	0.842	0.533	0.364	0.364	0.588	0.842
M							1.000	0.778	0.429	0.429	0.700	0.909
J								1.000	0.400	0.400	0.625	0.667
JU									1.000	0.667	0.333	0.429
A										1.000	0.500	0.429
S											1.000	0.800
O												1.000

**Table 6:** One-way ANOVA of Aquatic Insects at site (S1, S2, S3) in the Asan wetland during the study from November 2021 to October 2022.

Source of variation	*SS*	*Df*	*MS*	*F*	*P-value*	*F crit*
Between groups	126536.5896	2	63268.29479	1.967114143	0.155919713	3.284917651
Within groups	1061379.044	33	32163.00132			
Total	1187915.633	35				

SS Sum of Squares, *Df* Degrees of Freedom, *MS* Mean Square, *F* F-Statistic, *P-value* P-value, *F crit* Critical F-Value.

**Figure 2:**
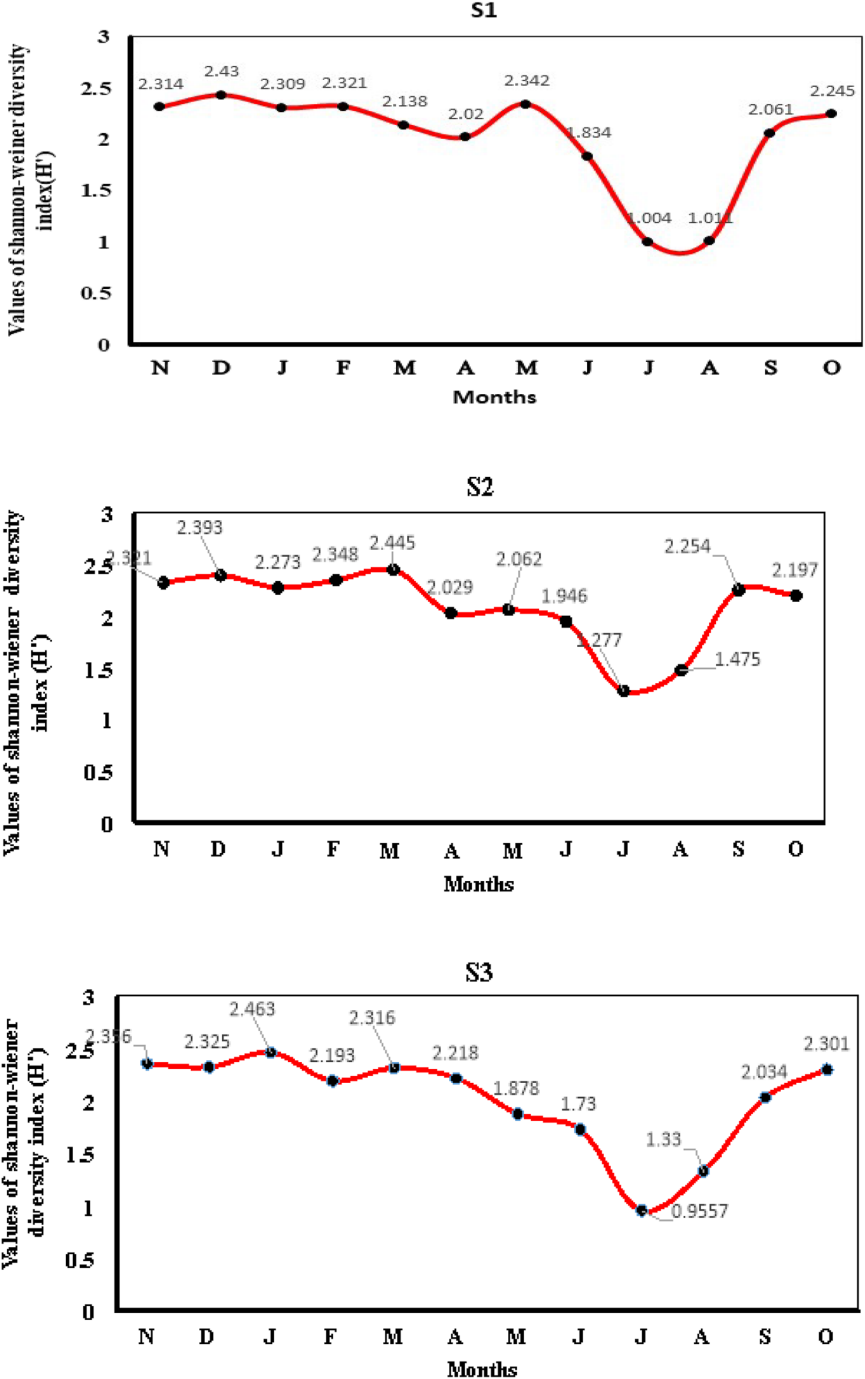
Monthly variations in the values of Shannon–Wiener diversity index(H’) at S1, S2, S3.

The diversity of the Asan Wetland, as measured by the Shannon–Wiener diversity index, varied across three sites (S1, S2, and S3) from November 2021 to October 2022. Months are labeled with their initial letters: "D" for December, "J" for January, "F" for February, "M" for March, and so forth, and so on, continuing until "O" for October. This facilitates easy interpretation of the monthly trends in the Shannon–Wiener diversity index, denoted as H′, across the three sampling sites (S1, S2, and S3) in the Asan Wetland as shown in Fig. 2. During the winter season, particularly in December, March, and January, the Shannon–Wiener diversity index values were relatively high, reaching 2.4, 2.445, and 2.461 at sites S1, S2, and S3, respectively. Conversely, the lowest values were observed in July at all sites throughout the study period, with values of 1.004, 1.277, and 0.9557 at sites S1, S2, and S3, respectively.

**Figure 3:**
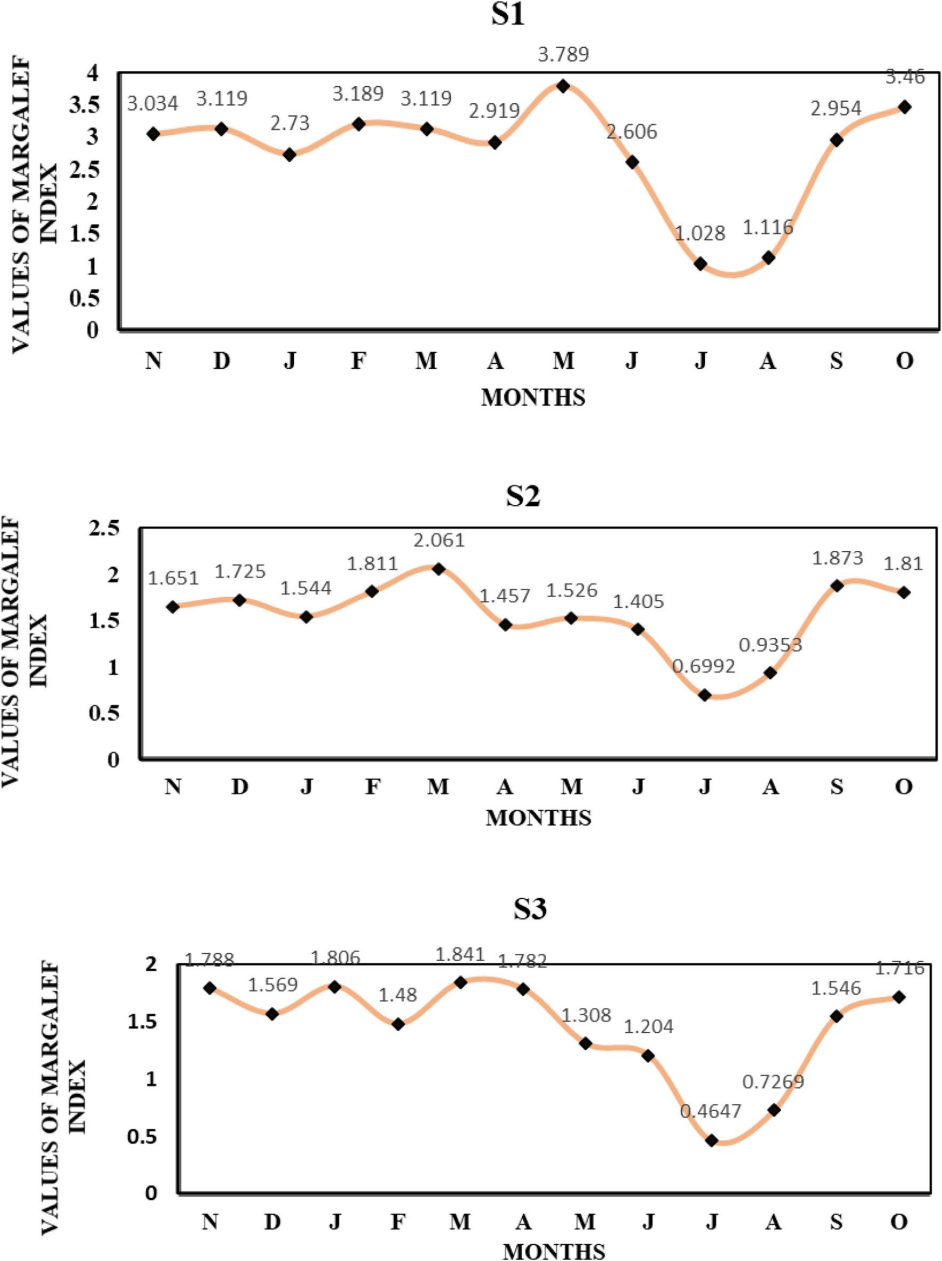
Monthly variations in the values of Margelef index at S1, S2, S3.

The Margalef Index values of the Asan Wetland from November 2021 to October 2022 at three sites (S1, S2 and S3) the Asan Wetland is given in Fig. 3. Each months are labelled with their respective initial letters: "D" for December, "J" for January, "F" for February, "M" for March, and so forth, continuing until "O" for October. At S1 Margelef index values were found high (3.789) in the month of May and low (1.028) in the month of July, whereas at S2 and S3, they were found high (2.061 and 1.841) in the month of March and low (0.6992 and 0.4647) in the month of July.

**Figure 4:**
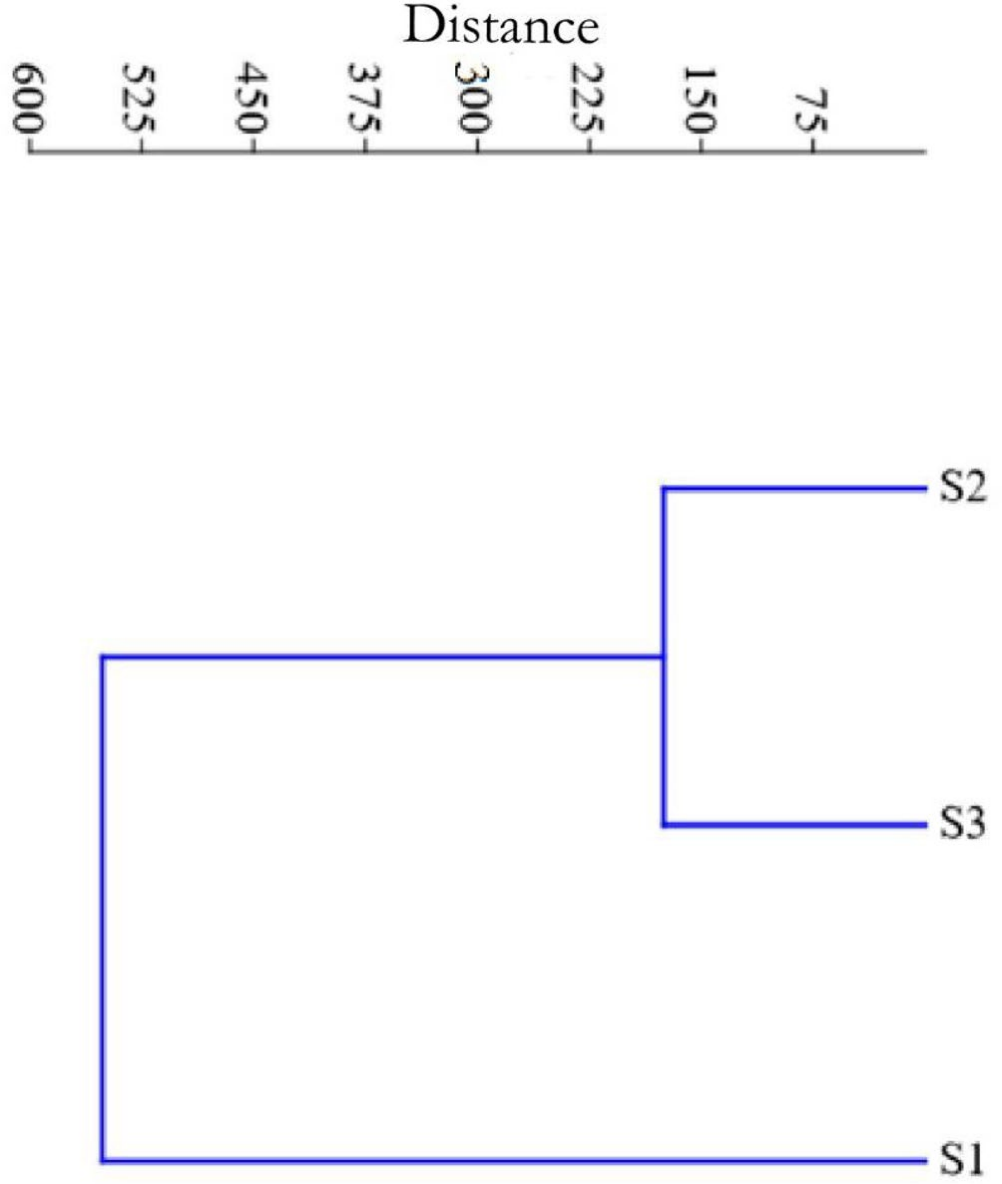
Cluster analysis of Aquatic Insects at site (S1, S2, S3) in the Asan wetland during the study from November 2021 to October 2022. (Average values).

Multivariate cluster analysis of aquatic insects of the Asan Wetland at S1, S2, S3 during the year 2021–2022 is presented in Fig. 4. The cladistic diagram showed that S2 and S3 were similar, whereas S1 was outgroup.

**Figure 5:**
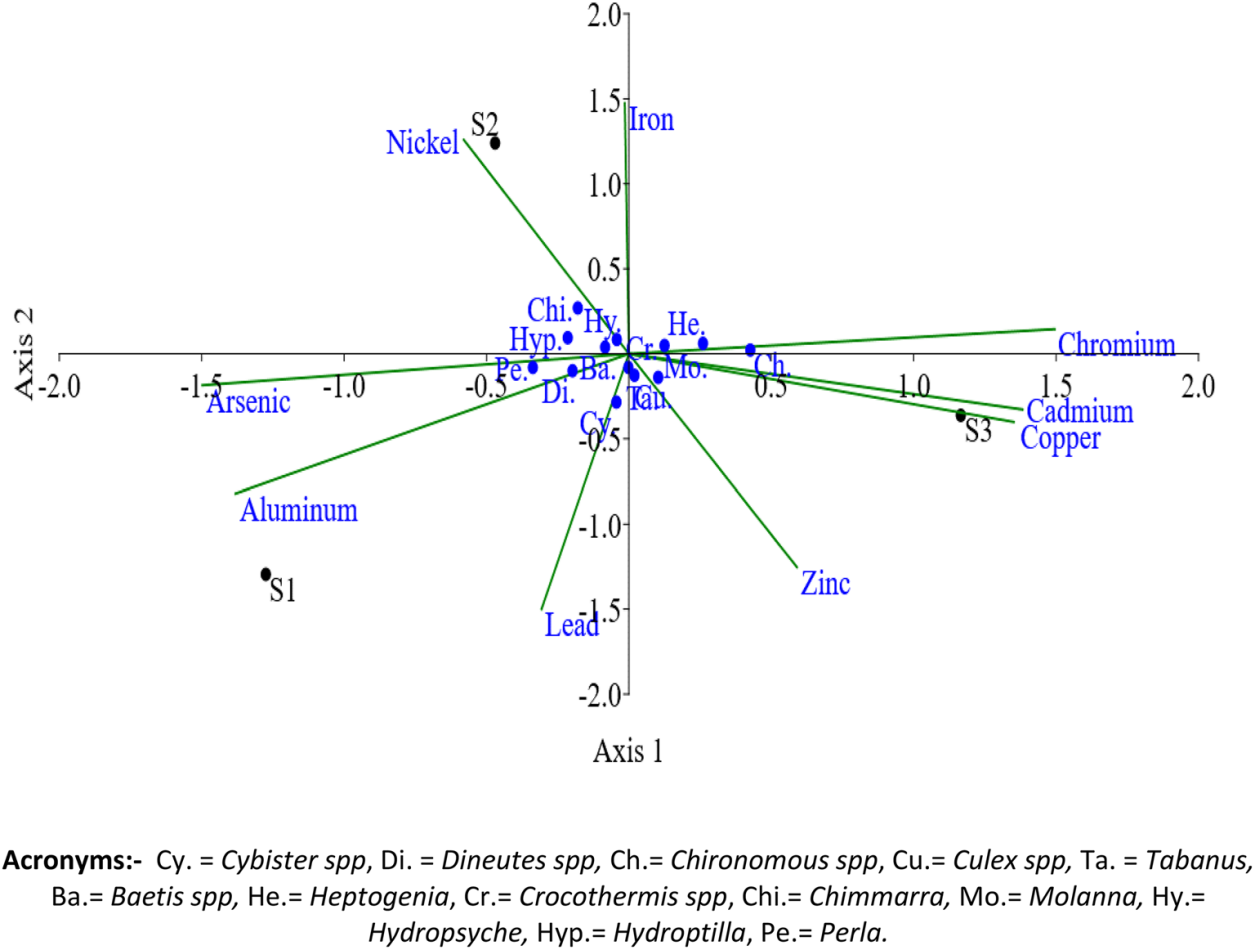
Canonical correspondence analysis (CCA) of Aquatic insects at the site (S1, S2, S3) in the Asan wetland during the study from November 2021 to October 2022 (Average values).

The CCA analysis presented in Fig. 5 depicts the relationship between heavy metal parameters and the abundance of aquatic insects in the Asan Wetland during a year-long study conducted from November 2021 to October 2022. Through CCA, the researchers explored how various detrimental heavy metal parameters affected the characteristic orders of aquatic insects at different sites (S1, S2, S3). The two axes of the CCA plot represents the variance at different axis and account for 69.01% and 30.99% of the variance, respectively, with eigenvalues of 0.032 and 0.014. The results indicated that the abundance of aquatic insects, represented by benthic orders such as Coeleoptera, Diptera, Ephemeroptera, Odonata, Trichoptera, and Plecoptera, was influenced by different heavy metals, including Cadmium, Copper, Arsenic, Iron, Lead, Chromium, Zinc, Nickel, and Aluminium. The S1 was represented by genera viz: Perla spp, Dineutes spp, Cybister spp, and Baetis spp governed by factors Arsenic, Aluminium, and Lead. The S2 was associated with genera like Hydroptilla spp, Chimerra spp, and Hydropsyche spp, and the major factors governing these were Nickel and Iron. The S3 was the most diverse as it was represented by most of the insect genera. The key genera associated with S3 were Culex spp, Chironomous spp, Heptagenia spp, Molanna spp, Tabanus spp and Crocothermis spp governed by factors Zinc, Copper, Cadmium and Chromium.

**Figure 6:**
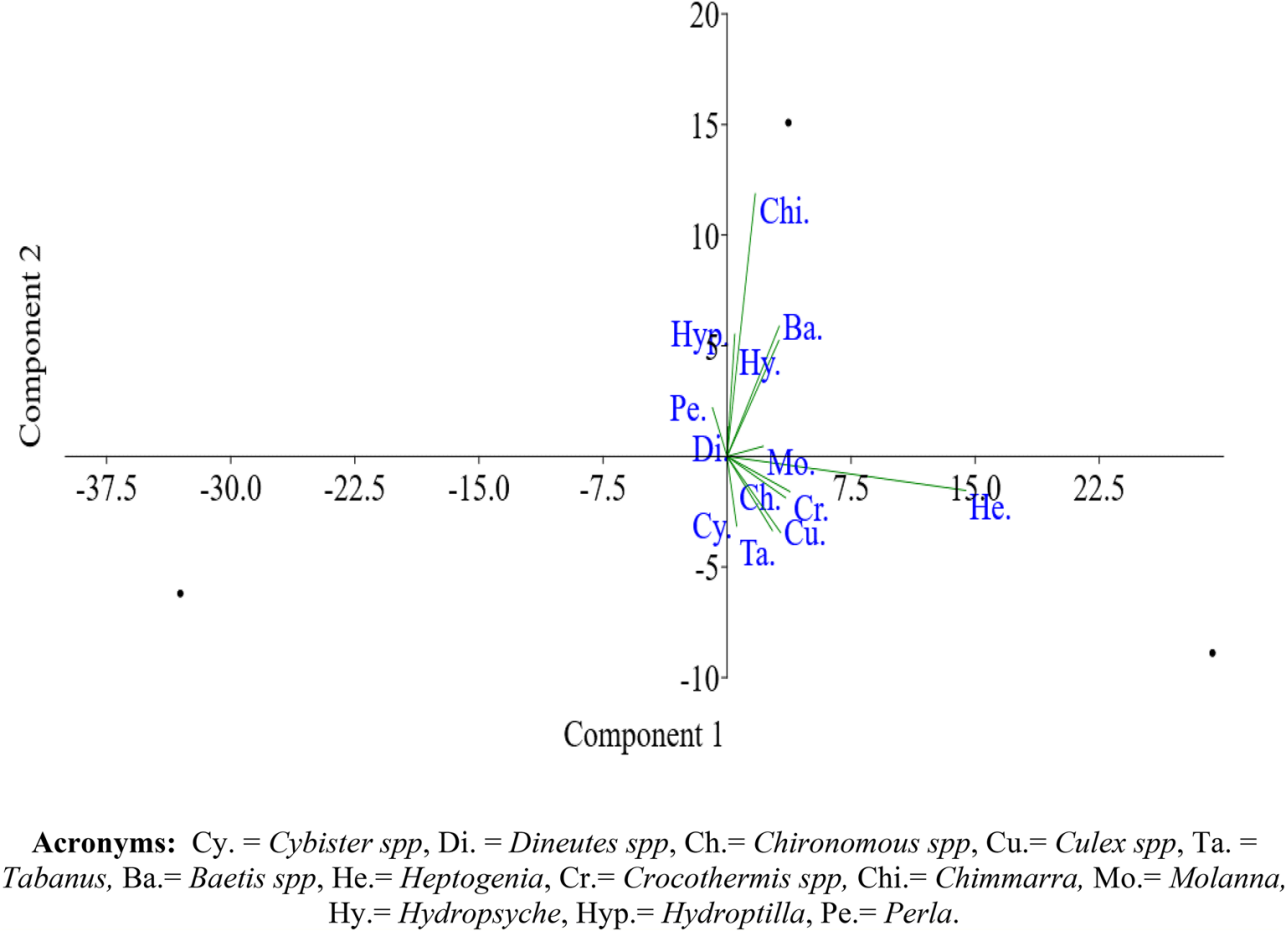
Principal component analysis (PCA) of Aquatic insects in the Asan wetland at the site (S1, S2, S3) during the study from November 2021 to October 2022 (Average values).

Multivariate Principal component analysis (PCA) was performed to study the abundance of various aquatic insects at sites (S1, S2, S3) in Asan Wetland during the study period (November 2021–October 2022) as represented in Fig. 6. The PC1(component 1) and PC2 (component 2) represented 59.26% and 40.73% of the variance with an eigen values of 7.70 and 5.29. The PCA plot thus formed suggested that S3 was the most diverse as it was represented by most of the aquatic insects (Cybister spp, Tabanus spp, Culex spp, Crocothermis spp, Chironomous spp, Heptagenia spp, Molanna spp and Hydropsyche spp) whereas S2 was represented by Baetis spp, Chimmarra spp, Hydroptilla spp, Dineutes spp and Perla spp and S1 represents the least genera of aquatic insects.

## Discussion

Heavy metals are characterized by their high density compared to other metals, and they possess toxic properties even at low concentrations^34,35^. Some of these elements are important for healthy metabolism, but when their concentration increases, they can also be toxic^36^.

In the Asan Wetland, it was found that all parameters demonstrated their highest average values during the monsoon season, followed by summer, and the lowest values during winter. These trends in various parameters align with the findings reported by^25^. According to their study, the elevated concentrations of heavy metals during summer and monsoon can be attributed to the higher evaporation rate caused by elevated temperatures^25,34,35^ also reported the highest accumulation of heavy metals in the pre-monsoon period for Gomti, Sabarmati, and Yamuna rivers. These results highlight the significant influence of seasonal dynamics and environmental factors on the concentration and distribution patterns of heavy metals within aquatic environments, including the Asan Wetland^25,37–39^ which supports the findings of this work.

As, it was found that the cadmium, copper, arsenic and Lead were maximum (0.0853 ± 0.0457), (0.192 ± 0.168), (1.03 ± 0.0783), (0.576 ± 0.173) in the month of August and minimum (0.0023 ± 0.00057), 0.0047 ± 0.00723), (0.0053 ± 0.0058), (0.006 ± 0.004) in January respectively.

Copper enters the water system through agricultural runoff, primarily originating from urban and agricultural areas. A similar pattern of maximum and minimum concentrations is observed for lead. Industries involved in activities such as paints and alloys release cadmium into the environment, which then mixes with sediments and particles. The input of cadmium into the aquatic environment occurs through the discharge of industrial waste, surface runoff, and deposition, as stated by IPCS (1992). In unpolluted areas, the average cadmium content in freshwaters ranges from less than 0.0001 to 0.00006 mg/L (IPCS, 1992).

A sewage from surrounding areas and industrial effluents as the primary sources of contamination, reporting the highest concentration of lead (0.98 mg/L) in the water of Haridwar river Ganga^40^. Iron levels showed a maximum value (5.74 ± 1.55) in September and a minimum value (1.41 ± 1.98) in January. The presence of iron in abundance can be attributed to anthropogenic activities such as urban industrial releases, domestic waste, and agricultural practices, as observed in studies conducted by^41^ in the Godavari River and other in the Gomti River, where anthropogenic effluents are discharged. Nickel and Aluminium exhibit similar trends to iron. The rapid discharge of sewage effluent into rivers like Ganga and Yamuna is the primary cause of increased Aluminium concentration. Industrial activities are the main sources of metals in rivers. Central Pollution Control Board (CPCB), provides the concentration ranges of various heavy metals, including Arsenic (As), Iron (Fe), Lead (Pb), Chromium (Cr), Nickel (Ni), Cadmium (Cd), Copper (Cu), Zinc (Zn), Mercury (Hg), and Manganese (Mn), observed in major rivers of the country during the year 2020.

The correlation between water hardness and the toxicity of metals to aquatic organisms has been widely acknowledged in scientific studies (Laws, 1981). It is well-documented that as water hardness increases, the bioavailability of metals like Cadmium (Cd), Copper (Cu), Nickel (Ni), Lead (Pb), and Zinc (Zn) in freshwater ecosystems generally decreases^42^. Water hardness plays a significant role in modulating the toxicity of cadmium to freshwater organisms. Cadmium is recognized as one of the most toxic heavy metals in freshwater environments. According to^42^, the permissible limits for cadmium in aquatic life range from 0.00066 to 0.002 mg/L. However, the measured cadmium concentrations at all sites exceeded the aforementioned values.

Lead is introduced into the aquatic environment through processes such as surface runoff and the deposition of lead particles from the air, as stated by IPCS (1989). The toxicity of lead to aquatic organisms varies depending on factors such as its availability, how it is taken up by organisms, and the sensitivity of different species. Generally, the early life stages of aquatic organisms are more susceptible to the toxic effects of lead. The toxicity of inorganic lead is heavily influenced by environmental factors including water hardness, pH levels, and salinity. In communities of aquatic invertebrates, there may be variations in sensitivity to lead contamination among different populations. Interestingly, populations from polluted areas have been observed to exhibit greater tolerance to lead compared to populations from non-polluted areas. According to^42^, the permissible limits for lead in aquatic life range from 0.0013 to 0.077 mg/L. However, the measured lead concentrations exceeded the prescribed limits.

In lentic ecosystem Macroinvertebrates constitutes an important component of tropic levels among aquatic biodiversity. These organisms play an important role in the processing of the ecosystem like nutrient cycling, metabolism of pollution, dispersion and secondary production of aquatic ecosystem^31^. While observing the benthic biota of the wetland, we have taken only the aquatic insects in this study. During this study, the maximum average aquatic insect’s density of all three sites was recorded during the month of January (613.5 ± 270.7ind./ m2), while the average minimum density was noticed for the month of August (71.8 ± 62.1ind./m2). It was only due to the presence of favourable environmental conditions but on the onset on monsoon season the perturbed ecological conditions and increase in heavy metal concentration restricted the growth of aquatic insects. Sharma and Rawat^21,22,43,44^, also observed that the macrozoobenthos showed a maximum population during winter and a minimum during monsoon season due to environmental conditions. A maximum mean density (332.78 ind/m2) of macroinvertebrates was observed at S3 and a minimum (192.9 ind./m2) at S1, whereas S2 is in between (292 ind/m2). Significant variations in macroinvertebrate density were observed among the three sampling sites. This disparity can be attributed to anthropogenic disturbances resulting from activities such as boating, tourist movement, oil leakage from motorboats, and other human-induced factors specifically at sampling site S1^43^. Some also noted a similar effect at this site concerning the dragonfly population (Odonota) in the Asan wetland. During the monsoon season, specifically in the months of July and August, the densities of macroinvertebrates were found to be at their lowest. However, starting from September, the densities began to increase and remained consistently high until the month of April. This pattern can be attributed to the significant sediment load transported by the Yamuna and Asan rivers during the monsoon period. As a result, there was a deposition of fine particles and disruptions in the littoral zone, leading to elevated concentrations of heavy metals in the water. These increased sedimentation and heavy metal levels in the rivers led to reduced water transparency, subsequently impacting primary production in the wetland. Consequently, food availability for macroinvertebrates was scarce during this period, compounded by the submergence of the littoral zone. The dominancy of insects (larvae and nymphs) as benthic fauna in the aquatic water bodies in winters has been documented in river Jhelum (Kashmir)^45^, Tons River^46^, and Hinyul stream, a tributary of river Ganga^47^. In this work, 13 genera belonging to 6 orders of macroinvertebrates were collected. These genera are derived from different phyla (Annelida, Mollusca and Arthopoda). The spatiotemporal variations in diversity and density among the macroinvertebrates depend on the physio-chemical characteristics of water, heavy metals concentration availability of suitable habitat and their biological interactions. The results of correlation between density of macroinvertebrates and heavy metal parameter indicates that macroinvertebrates show negative correlation with all the heavy metals; when concentration of heavy metals increases in monsoon season the macroinvertebrates shows decrease in density and diversity.

The maximum richness and density of benthic fauna were observed during the winter season, which is favorable due to lower temperatures^21^ and higher levels of dissolved oxygen^48^. These environmental conditions support the thriving population of benthic organisms. Additionally, the availability of phytoplankton as a food source contributes to the increased density. On the other hand, during the rainy season, a decline in the density of benthic fauna was observed. This decline can be attributed to factors such as surface runoff containing untreated sewage, dilution effects, and the presence of contaminants. These factors increase the load of suspended solids, reduce water transparency, and result in increased water flow, ultimately affecting the distribution of benthic organisms in the Parbati River. Various experts in the field have reported similar findings^27,28,49,50^.

Table 3 shows the overall average dissimilarity of aquatic insects in the Asan wetland. It indicates that the mean average dissimilarity of aquatic insects at three selected sites of Asan wetland was 23.3% whereas the average dissimilarity in abundance was 22.17%. This table also shows the mean and abundance of aquatic insects at three different sites. Determination of the similarity coefficient is a tool to know how the fauna is similar or dissimilar in different months. By knowing this we can estimate the detrimental factors responsible for this. The coefficient of Similarity of macrozoobenthos/aquatic insects between different month during 2021–2022 is presented in the Table 4. The high values of similarity index were observed for the month of November–December (1.0) and minimum for July–August (0.143). It indicated that the environmental conditions were conducive during winter and disturbed during monsoon. Similarly, high values of similarity index (0.98–1.0) during winter months are also reported in the Nayar river system^22^.

Shannon–Wiener diversity index is the most preferred index for calculating faunal diversity in ecological studies. At sites S1, S2 and S3, its peak in December (2.43), March (2.445), January (2.463), respectively and was highest at S3 showed most commonly preferred environment by most aquatic insects due to moderate values of all detrimental parameters. Whereas, the lowest value in July (1.004, 1.277, 0.9557) at all sites respectively and was lowest at S3 among three sites indicates towards availability of less species due to disturbed ecological conditions (Fig. 2). In^21,22^ the range of this index is from 0 to 5, but generally it falls between 1.5 and 3.5. A value above 3 shows an established ecosystem, and below 1 indicates habitat degradation and pollution. The value of Margalef diversity index was between 3.789 and 1.841 (Fig. 3). During the investigation, Margelef index values were found high (3.789) in the month of May and low (1.028) in the month of July at S1, whereas at sites S2 and S3, they were found high (2.061 and 1.841) in the month of March and low (0.6992 and 0.4647) in the month of July. The computation of this index is based on the count of species or species richness observed at various sampling sites during different sampling occasions^21^. In the Semenyih River, located in peninsular Malaysia, the species richness, as indicated by the Margalef index value, exhibited a range of 0.08 to 1.90 across seven distinct sampling stations.

The multivariate cluster analysis is performed between three different sites to identify the homogeneous sites. The cladistic diagram (Fig. 4) between three different sites showed that S2 and S3 were similar, whereas S1 was outgroup. Negi and Mamgain^47^ also applied cluster analysis for macrozoobenthos to identify homogeneous groups between different months^21,22^.

The canonical correspondence analysis is an important statistical tool under multivariate analysis to find out the detrimental heavy metal parameters for the growth of a particular population at three different sites. In the present analysis, CCA was performed to investigate the effect of different detrimental heavy metal parameters on the characteristic orders at different sites, which may be favourable or unfavourable for certain species.

The CCA plot (Fig. 5) suggested that the S3 was the most diverse, followed by the S1 and S2. The association of various genera (Culex spp, Chironomous spp, Heptagenia spp, Molanna spp, Tabanus spp and Crocothermis spp) with heavy metals such as Zinc, Copper, Cadmium and Chromium at S3 suggested that these are more tolerable towards the associated heavy metals and sensitive to rest of the heavy metals (Nickel, Aluminium, Arsenic, Lead and Iron). The genera found at S1 were more resistant to Arsenic, Aluminium and Lead heavy metals, whereas genera associated with S2 showed resistance towards only two heavy metals (Nickel and Iron). Researchers used the CCA to study benthic macroinvertebrates at different sites in the Bhagirathi River^51^ and benthic population in the Western Nayar river of Garhwal Himalaya^20^. A study conducted by^52^ investigated the correlation between fauna and environmental data matrices using Canonical Correspondence Analysis (CCA), and their results indicated a significant relationship. In a separate study in Tajan River, Iran. In another study, CCA was utilized to assess the impact of abiotic parameters on benthic biota^53^. Their findings highlighted those factors such as dissolved oxygen, pH, water temperature, and turbidity significantly influenced the distribution of benthos. Similarly^25^ employed CCA to examine the spatial distribution of benthic invertebrate communities in response to surrounding environmental variables in the Gomti River. They found it suitable to conclude that land use pattern is influential in altering water quality and, hence the distribution of benthic insects.

## Conclusion

Various studies explain that heavy metals are introduced to aquatic ecosystems through natural processes and anthropogenic activities like industrial processes, mining, agriculture, urban runoff, and the use of metal-containing compounds like fertilizers and pesticides.

- Impact on Ecological Balance: The elevated concentrations of heavy metals, especially during summer and monsoon seasons, are attributed to higher evaporation rates and environmental factors. This is in line with findings from other rivers like Gomti, Sabarmati, and Yamuna, which reported the highest accumulation of heavy metals in the pre-monsoon period .
- Effect on Biodiversity: The presence of heavy metals negatively affects the aquatic ecosystem, particularly impacting the distribution and population of macroinvertebrates like aquatic insects. The study found a negative correlation between macroinvertebrates and heavy metal concentrations; as the concentration of heavy metals increases, particularly in the monsoon season, there is a decrease in the density and diversity of macroinvertebrates ​​.

This study demonstrates a seasonal variation in heavy metal concentration in the Asan Wetland, with higher levels observed during the monsoon due to factors such as agricultural runoff, industrial discharges, and increased sediment transport. Notably, the monsoon season's unfavourable conditions, including soil erosion and poor waste management, significantly contribute to this escalation. Conversely, a decrease in heavy metal concentration is observed in winter, underscoring the influence of seasonal dynamics on wetland ecology.

Site-specific variations were also a focal point of our findings. For instance, at Site S1, a popular location for boating and tourism, a decline in the density of aquatic insects and an increase in heavy metals were observed, likely due to oil spillage from boats. In contrast, Site S3 exhibited a higher density of aquatic insects and lower heavy metal concentrations, attributed to its shallow water depth and better water flow, which foster favourable conditions for benthic biota.

These findings highlight the profound negative impact of heavy metal pollution on the distribution and density of aquatic insects in wetlands. It underscores the critical need for effective monitoring and management of heavy metal contamination to safeguard freshwater ecosystems and the vital ecological services provided by aquatic insects. The study also points to the necessity of further research and conservation efforts to fully understand the long-term effects of such pollution and to develop comprehensive strategies for mitigating its impact on aquatic ecosystems. Thus, this research serves as a call to action for enhanced environmental stewardship and policy-making geared towards preserving the integrity and biodiversity of wetland ecosystems.

## Data Availability

The datasets obtained and analyzed in the current study are available from the corresponding author on reasonable request.
